# Dance‐Based Interventions for Mental Health: A Comprehensive and Quality‐Sensitive Umbrella Review of Definitions, Measurement Practices, and Evidence Fragmentation

**DOI:** 10.1111/nyas.70358

**Published:** 2026-08-08

**Authors:** Xinyu Dou, Xu Zhu

**Affiliations:** ^1^ The University of Hong Kong Hong Kong China; ^2^ University of Auckland Auckland New Zealand

**Keywords:** dance, intervention, mental health, mental well‐being, umbrella review

## Abstract

Dance‐based interventions are increasingly used to support mental health and well‐being across clinical, community, and educational contexts. However, the review‐level evidence remains conceptually and methodologically fragmented. This quality‐sensitive umbrella review synthesized 90 review articles identified across five databases. Systematic reviews and meta‐analyses were appraised using AMSTAR 2, while scoping, narrative, literature, and clinical reviews were assessed using adapted review‐appraisal criteria. Findings from systematic reviews and meta‐analyses were used primarily to interpret effectiveness evidence, whereas nonsystematic reviews were used to map conceptual definitions, practice traditions, and measurement issues. The synthesis showed that the field is fragmented at three interrelated levels. First, intervention labels and delivery models are inconsistent, ranging from clearly defined dance/movement therapy to broad and underspecified uses of dance. Second, effectiveness is conceptualized differently across intervention traditions, with therapy‐oriented reviews emphasizing symptom reduction and non‐therapy‐oriented reviews foregrounding well‐being, vitality, social connection, and embodied experience. Third, outcome assessment relies predominantly on generic psychological scales, with limited use of movement‐sensitive or dance‐specific measures. Quality‐sensitive evidence suggests that dance‐based interventions show promise for well‐being, social connection, emotional regulation, and selected symptom outcomes, but confidence remains limited by low review quality, heterogeneous populations, inconsistent intervention reporting, and insufficient attention to primary‐study overlap. Future research should move beyond asking whether dance works in general and instead specify what kind of dance, for whom, through which mechanisms, and according to which model of mental health and well‐being.

## Graphical abstract

This study is a comprehensive, quality‐sensitive umbrella review that examines how dance‐based interventions for mental health are defined and categorized across the review literature, how effectiveness is conceptualized and measured, what evidence patterns emerge across populations and moderators, and what conceptual and methodological limitations continue to weaken the field.

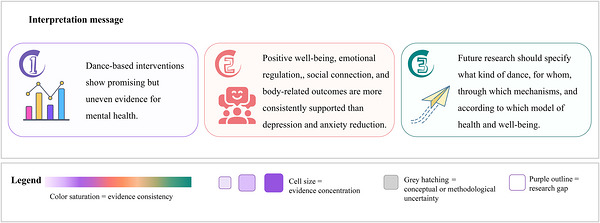

## 1 Introduction

### 1.1 Previous Reviews

As interest has grown in integrated approaches to health that attend to both physical and psychological well‐being across diverse groups, dance has increasingly been examined for its therapeutic value. Existing reviews, however, have largely addressed this topic from relatively narrow and separate perspectives. Some reviews focus on dance's impact on physical function rehabilitation [1, 2, 3, 4, 5, 6, 7], examining outcomes such as motor improvement [8, 9, 10, 11, 12, 13, 14, 15, 16], and cardiovascular health [17, 18]. Others situate dance within broader categories, such as the arts [19, 20, 21, 22, 23, 24, 25, 26, 27, 28, 29], mind−body therapies [30], creative activities [31], or physical activity [32, 33], with some even conducting economic evaluations of arts intervention [34]. In addition, a subset of reviews provides a broad historical overview of dance/movement therapy as a field [35, 36].

Existing review literature has undoubtedly advanced the understanding of dance in relation to health and well‐being. However, this literature has grown in disconnected silos, often organized around specific populations, diagnoses, or intervention traditions rather than around shared conceptual and methodological questions. As a result, an important problem remains insufficiently addressed: not only whether dance‐based interventions are beneficial, but how dance is being defined, what counts as effectiveness, and whether prevailing measurement approaches are adequate for capturing dance as an embodied, relational, and artistic practice. Even where reviews report positive effects, it often remains unclear whether differences across findings reflect true variation in intervention impact or deeper inconsistencies in intervention description, outcome framing, and review logic. Furthermore, to date, no umbrella review has focused specifically on dance as a core intervention for mental health outcomes. While Ciacchella et al. [37] included dance in their umbrella review, they treated it as one of many activities, without isolating its unique contribution or examining dance‐specific mechanisms and modalities.

### 1.2 Present Study

This study is not a second‐order meta‐analysis of intervention effects. Rather, it is a comprehensive, quality‐sensitive umbrella review that synthesizes review‐level evidence to examine conceptualization, measurement practices, population and outcome patterns, and evidence fragmentation in dance‐based mental health research. This umbrella review, therefore, aims to move beyond a simple summary of positive or negative outcomes. It examines how dance‐based interventions for mental health are defined and categorized across the review literature, how effectiveness is conceptualized and measured, what evidence patterns emerge across populations and moderators, and what conceptual and methodological limitations continue to weaken the field. By synthesizing 90 reviews across diverse traditions, this study seeks to clarify not only the current evidence base but also why that evidence remains fragile and uneven. The review is guided by the following research questions.

**RQ1**: How are dance‐based interventions for mental health defined and categorized across the review literature?
**RQ2**: How is effectiveness conceptualized and measured in this field?
**RQ3**: What patterns of evidence emerge across populations, outcomes, and moderators, and how robust are these patterns?
**RQ4**: What conceptual and methodological limitations continue to constrain the interpretability and applicability of the current evidence base?

## 2 Method

This study was designed as a quality‐sensitive umbrella review of review‐level evidence rather than a second‐order meta‐analysis. Unlike primary research, which generates original data, or conventional reviews that synthesize primary studies on a narrowly defined question, an umbrella review integrates findings from existing reviews to generate a broader understanding of a complex field [38, 39]. This design was appropriate for the present study because the literature on dance‐based interventions for mental health comprises a sufficient number of component reviews examining different populations, intervention traditions, outcome domains, and methodological issues.

The aim was not to statistically reanalyze pooled effect sizes reported in previous meta‐analyses, but to synthesize how dance‐based interventions for mental health have been conceptualized, measured, and evaluated across the review literature. The field includes not only systematic reviews and meta‐analyses of intervention effects, but also scoping, narrative, literature, and clinical reviews that have shaped how dance‐based practices are defined and interpreted. To avoid treating all review types as equivalent, systematic reviews and meta‐analyses were used primarily to interpret evidence of intervention effects, whereas nonsystematic reviews were used primarily to map conceptual definitions, intervention traditions, measurement practices, and emerging debates.

Accordingly, this review synthesized 90 prior reviews to advance understanding in four areas: (a) conceptualizations of dance‐based interventions and their proposed processes of change; (b) intervention forms, delivery models, and measurement approaches; (c) population characteristics and potential moderators of intervention outcomes; and (d) review‐level evidence on mental health outcomes, ranging from symptom reduction to positive well‐being, while also identifying conceptual and methodological gaps that constrain interpretation.

### 2.1 Study Identification

A search for reviews on dance/movement intervention was performed using five computer databases (PsycINFO, PsycArticles, PubMed, Web of Science, Scopus) in November−December 2025. The search terms used pertained to dance/movement intervention ((danc* OR movement OR “creative movement” OR “dance movement therap*” OR “dance‐movement therapy” OR “dance/movement therapy” OR “dance‐movement” OR “dance/movement” OR “dance therap*” OR “therapeutic dance” OR “community dance” OR “mindful movement” OR “somatic practice*” OR “authentic movement” OR “dance intervention*” OR “movement intervention*” OR “dance‐movement intervention” OR “dance/movement intervention” OR “dance‐based” OR “movement‐based” OR “performing arts”)) AND ((“mental well‐being” OR “psychological well‐being” OR “subjective well‐being” OR “emotional well‐being” OR “mental health” OR “psychological health” OR “life satisfaction” OR flourishing)) AND ((“review of literature” OR “literature review” OR meta‐analysis OR “meta analysis” OR “systematic review”)).

### 2.2 Selection of Studies

Eligibility for inclusion in the umbrella review was determined according to predefined criteria. Studies were considered eligible if they met the following conditions:

- They were qualitative, quantitative, mixed‐methods, scoping, systematic reviews, or meta‐analyses.
- They focused primarily on dance‐based interventions, or reported dance‐specific findings separately.
- They examined at least one mental health or mental well‐being outcome.
- The full text was available in English.

A study was excluded if it met one of the following conditions:

- It was a nonreview article.
- It was a primary study or a study examining secondary data.
- It involved irrelevant interventions, such as pure music therapy, etc.
- It reported findings on dance that could not be separated, or there were not enough dance studies included to conclude any outcome measure.
- It was a review only reporting physiological outcomes.
- The full text was not available.
- It was a duplicate study.
- It was a retraction article.

Included reviews were classified into two evidence‐use categories. The first category comprised systematic reviews and meta‐analyses, including mixed systematic review and meta‐analysis papers. These reviews were considered eligible for quality‐sensitive interpretation of effectiveness evidence. The second category comprised scoping reviews, narrative reviews, literature reviews, and clinical reviews. These reviews were not used to support strong claims about intervention effectiveness, but were retained because they provided important information about intervention definitions, conceptual traditions, practice contexts, and measurement gaps. This distinction was maintained throughout the synthesis.

### 2.3 Coding

A coding scheme was developed to record both substantive and methodological features of the included reviews.

- Basic bibliographic information (author(s), publication years, title of study)
- Review design (e.g., literature review, systematic review, meta‐analysis, mixed review, scoping review)
- Use of preferred reporting items for systematic review, meta‐analysis, and mixed review (PRISMA) (yes, no)
- Use of inclusion/exclusion criteria for selection of studies (yes, no)
- Conceptualization of dance intervention
- Measurement of mental health‐related outcomes
- Moderating factors identified

Two researchers coded each review independently. Overall agreement reached 91.6%, and any discrepancies were resolved through discussion.

### 2.4 Research Quality Evaluation

Methodological quality was appraised separately according to review type. Systematic reviews, meta‐analyses, and mixed systematic review and meta‐analysis papers were assessed using AMSTAR 2. The 16 AMSTAR 2 items were coded as “yes,” “partial yes,” “no,” or “not applicable,” following AMSTAR 2 guidance. Overall confidence ratings were assigned as high, moderate, low, or critically low according to the number and severity of weaknesses in critical and noncritical domains. Because AMSTAR 2 is not designed for nonsystematic review formats, scoping, narrative, literature, and clinical reviews were assessed using adapted criteria focused on five dimensions: clarity of review aim, transparency of search or source selection, clarity of inclusion scope, transparency of synthesis procedure, and coherence between evidence and conclusions. These adapted ratings were used only to support quality‐sensitive interpretation and were not treated as equivalent to AMSTAR 2 ratings.

### 2.5 Synthesis Strategy

The synthesis proceeded in three stages. First, a conceptual synthesis was conducted to identify how dance‐based interventions were defined, labeled, and categorized, as well as how effectiveness or healing was framed across different review traditions. Second, an evidence‐mapping approach was used to summarize patterns across populations, mental health outcomes, and potential moderators. Third, all interpretations were made in a quality‐sensitive manner. Greater interpretive weight was assigned to higher‐quality systematic reviews and meta‐analyses when discussing effectiveness claims, while narrative, literature, and scoping reviews were used primarily to identify conceptual trends, intervention descriptions, and emerging methodological issues. In this way, the umbrella review aimed to retain the breadth of the field without collapsing important distinctions in evidential strength.

## 3 Results

### 3.1 Characteristics and Methodological Quality of Included Studies

**FIGURE 1:**
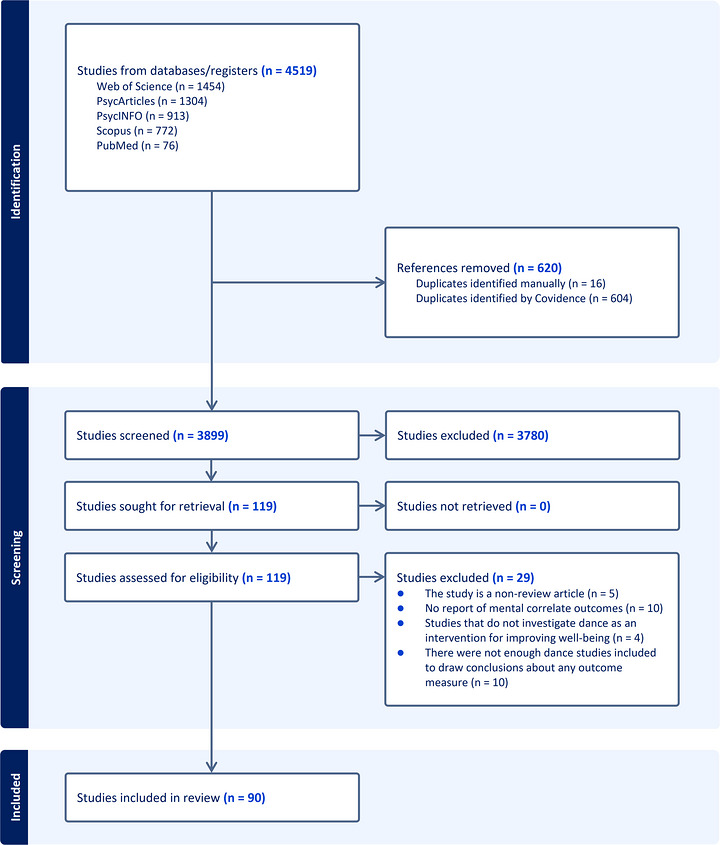
PRISMA diagram.

The umbrella review included 90 reviews (Figure 1): 46 systematic reviews, 24 mixed systematic review/meta‐analysis papers, 10 meta‐analyses, 4 scoping reviews, 4 literature reviews, 1 narrative review, and 1 clinical review. Systematic reviews, meta‐analyses, and mixed review/meta‐analysis papers were used primarily to interpret effectiveness evidence, whereas scoping, narrative, literature, and clinical reviews were used mainly to support conceptual and methodological mapping (Table 1).

**TABLE 1:** Summary of reviews.

Included review	Review design	Use of PRISMA (√/×)	Explicit study selection criteria reported (√/×)	Quality rating	Intervention focus
Abu‐Odah et al. (2024) [46]	Mixed	√	√	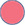	Dance/movement therapy (DMT)
Acolin (2016) [61]	Systematic review	×	√	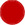	Dance/movement therapy (DMT)
Allred (2023) [125]	Literature review	×	×	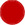	Dance/movement therapy (DMT)
Oepen (2025) [56]	Systematic review	√	√	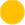	Creative arts therapies
Guerrero Ariza and Bräuninger (2025) [62]	Systematic review	×	√	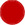	Dance/movement therapy (DMT)
Bai et al. (2022) [64]	Mixed	√	√	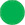	Plaza dancing
Aithal et al. (2021) [43]	Systematic review	√	√	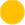	Dance movement psychotherapy (DMP)
Koch et al. (2019) [49]	Meta analysis	√	√	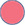	Dance/movement therapy (DMT)
Amonkar et al. (2021) [44]	Systematic review	√	√	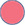	Dance intervention and DMT
Barnish and Nelson‐Horne (2023) [52]	Systematic review	√	√	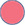	Dance intervention
Chen et al. (2024) [67]	Systematic review	√	√	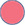	Dance for health and DMT
Bell et al. (2025) [50]	Systematic review	√	√	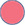	DMT/creative dance therapy
Bradt et al. (2015) [73]	Meta analysis	√	√	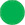	Dance/movement therapy (DMT)
Búrány and Tarkó (2025) [95]	Systematic review	√	√	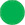	Dance/movement therapy (DMT)
Burkhardt and Brennan (2012) [96]	Systematic review	√	×	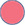	Dance intervention
Carapellotti et al. (2020) [53]	Mixed	√	×	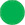	Dance intervention
Chappell et al. (2021) [66]	Systematic review	√	×	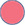	Dance intervention
Cheng et al. (2024) [67]	Mixed	√	√	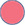	Dance intervention
Chiang et al. (2019) [126]	Systematic review	√	√	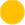	Dance intervention
Christopher et al. (2025) [47]	Mixed	√	√	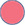	Dance therapy
De Jesus et al. (2020) [54]	Systematic review	√	√	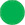	Dance/movement therapy (DMT)
Du et al. (2025) [127]	Scoping review	√	√	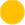	Dance intervention
Dumitru (2025) [55]	Scoping review	√	√	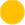	Dance intervention
Fan et al. (2025) [92]	Meta analysis	√	√	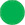	Dance intervention
Fatkulina et al. (2021) [74]	Systematic review	√	×	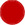	Dance/movement therapy (DMT)
Lee et al. (2025) [128]	Clinical review	×	×	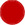	Dance/movement therapy (DMT)
Fonseca et al. (2025) [108]	Systematic review	√	√	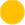	Dance intervention
Jiménez et al. (2019) [129]	Systematic review	√	√	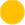	Dance/movement therapy (DMT)
Guo et al. (2020) [93]	Meta analysis	√	√	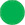	Dance
Huang et al. (2023) [122]	Mixed	√	√	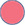	Dance/movement therapy (DMT)
Huang (2025) [110]	Systematic review	√	√	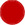	Dance/movement therapy (DMT)
Jaldin et al. (2025) [87]	Mixed	√	√	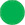	Dance intervention
Karkou et al. (2023) [48]	Meta analysis	√	√	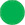	Dance/movement therapy (DMT)
Karkou et al. (2019) [130]	Mixed	√	√	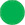	Dance/movement therapy (DMT)
Kiepe et al. (2012) [79]	Systematic review	√	√	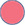	DMT and social dance
Kleinlooh et al. (2021) [131]	Systematic review	√	√	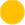	Dance/movement therapy (DMT)
Koch et al. (2014) [111]	Meta analysis	√	√	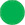	DMT and dance
Kwok et al. (2016) [41]	Mixed	√	√	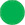	Dance
Lavania and Ballal (2026) [132]	Systematic review	√	√	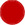	Dance/movement therapy (DMT)
Levine and Land (2016) [133]	Systematic review	×	√	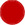	Dance/movement therapy (DMT)
Liang and Bryant (2024) [104]	Systematic review	√	√	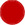	Dance/movement therapy (DMT)
Liao et al. (2025) [42]	Mixed	√	√	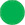	Dance
Li et al. (2021) [103]	Meta analysis	√	√	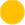	Dance
Liu et al. (2021) [97]	Mixed	√	√	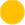	Dance intervention
Liu et al. (2023) [134]	Systematic review	√	√	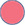	Latin dance
Liu et al. (2025) [135]	Systematic review	√	√	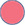	Dance
Liu et al. (2022) [136]	Systematic review	√	√	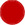	Dance
Santos et al. (2020) [115]	Systematic review	√	√	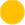	Dance
Mala et al. (2012) [114]	Scoping review	×	√	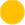	Dance/movement therapy (DMT)
Li et al. (2025) [75]	Systematic review	√	√	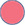	Square dance
Wu et al. (2021) [117]	Mixed	√	√	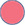	Dance intervention
Lu et al. (2025) [45]	Mixed	√	√	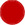	Dance
Luo et al. (2024) [81]	Mixed	√	√	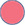	Dance
McQuade and O'Sullivan (2024) [137]	Systematic review	√	√	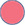	Dance
Meekums et al. (2015) [80]	Systematic review	√	√	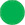	Dance/movement therapy (DMT)
Mo et al. (2025) [83]	Meta analysis	√	√	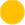	Dance
Morateli et al. (2023) [105]	Systematic review	√	√	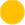	Dance intervention
Nelson et al. (2023) [76]	Mixed	√	√	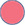	Dance
Niranjan et al. (2022) [138]	Systematic review	√	√	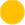	Dance
Noice et al. (2014) [98]	Systematic review	×	√	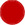	Dance
Ren and Xia (2013) [63]	Mixed	√	√	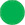	Dance
Patel et al. (2025) [77]	Systematic review	√	√	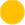	Dance
Olvera (2008) [94]	Literature review	×	√	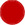	Culture dance
Ou et al. (2022) [99]	Systematic review	√	√	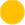	Square dance
Podolski et al. (2023) [88]	Mixed	√	√	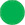	Dance intervention
Prudente et al. (2024) [82]	Mixed	√	√	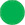	Dance intervention
Zhang and Wei (2024) [109]	Literature review	√	√	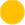	Dance/movement therapy (DMT)
Rao et al. (2019) [84]	Narrative review	√	√	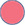	Dance (Bharatanatyam)
Ronzi et al. (2018) [100]	Systematic review	√	√	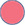	Dance
Ritter and Low (1996) [120]	Meta analysis	×	√	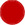	Dance/movement therapy (DMT)
Rudolph et al. (2018) [78]	Systematic review	√	√	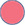	Ballroom dance
Salihu et al. (2021) [121]	Mixed	√	√	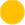	Dance intervention
Şanlı et al. (2025) [112]	Systematic review	√	√	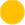	Dance/movement therapy (DMT)
Schwender et al. (2018) [85]	Systematic review	√	√	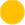	Dance intervention
Sánchez‐Alcalá et al. (2023) [89]	Mixed	√	√	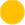	Dance
Sheppard and Broughton (2020) [90]	Systematic review	√	√	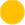	Dance
Shim et al. (2021) [91]	Systematic review	√	√	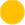	Dance intervention
Sivvas et al. (2015) [106]	Literature review	×	×	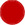	Dance
Yang et al. (2025) [139]	Meta analysis	√	√	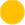	Dance
Son and Kwon (2024) [86]	Systematic review	√	√	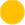	Dance
Strassel et al. (2011) [113]	Systematic review	√	√	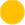	Dance therapy
Takahashi and Kato (2023) [57]	Systematic review	√	√	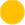	Dance/movement therapy (DMT)
Tao et al. (2022) [140]	Systematic review	√	√	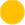	Dance
Thompson et al. (2026) [107]	Scoping review	√	√	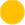	Dance/movement therapy (DMT)
Tomaszewski et al. (2023) [141]	Systematic review	√	√	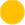	Dance therapy
Vaseinasrabadi and Desouza (2026) [116]	Systematic review	√	√	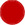	Dance
Wang et al. (2021) [123]	Mixed	√	√	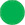	Dance intervention
Yan et al. (2024) [118]	Mixed	√	√	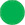	Dance intervention
Yoong et al. (2024) [101]	Mixed	√	√	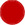	Dance
Zhang et al. (2025) [102]	Mixed	√	√	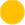	Dance intervention

*Note*: Mixed = systematic review and meta‐analysis.

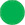
 *= High*; 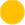 *= Moderate*; 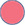 *= Low*; 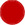 *= Critical low*.

“√” indicates that the review used PRISMA or reported explicit study selection criteria, whereas “×” indicates that it did not.

Methodological quality varied substantially across review types. AMSTAR 2 appraisal showed that most systematic reviews and meta‐analyses were rated as low or critically low. The most frequent limitations concerned incomplete reporting of duplicate study selection and data extraction, inadequate risk‐of‐bias assessment of primary studies, limited reporting of primary‐study funding sources, insufficient consideration of risk of bias when interpreting findings, and limited assessment of publication bias in meta‐analyses. These limitations do not invalidate the evidence base, but they require cautious interpretation of effectiveness claims.

A pattern was evident across the higher‐quality reviews; they tended to focus on more clearly specified populations and better‐defined intervention forms. For example, reviews with clearer intervention definitions often focused on dance/movement therapy, plaza dancing, square dance, or population‐specific dance interventions. In contrast, reviews using broad labels such as “dance” or “dance intervention” without sufficient detail on form, delivery, or practitioner role were more difficult to interpret.

### 3.2 Intervention Labels and Delivery Models

The reviews included reveal that dance‐based interventions assume diverse forms, varying in purpose and content. However, the majority of reviews fail to provide clear, operational definitions of the dance activities under investigation [40]. For example, Kwok et al. [41] and Liao et al. [42] examined the effects of dance on mental health outcomes but provided insufficient detail on the type, structure, or delivery of the dance activities involved. This underspecification is not merely a limitation of individual reviews; it reflects a deeper problem rooted in the primary studies themselves. Existing reviews consistently note that primary studies rarely provide detailed descriptions of their intervention protocols [43, 44, 45], and specific intervention elements often remain unspecified [43, 46]. Consequently, reviewers are left with insufficient raw material from which to derive clear categorizations, perpetuating a cycle of conceptual ambiguity that precludes accurate assessment of how different forms of dance intervention may differentially affect mental health outcomes. As Christopher et al. [47] argue, future research must provide more detailed reporting of how dance interventions are delivered to enable replication, meaningful comparison across studies, and ultimately, translation into practice.

Despite these limitations, a fundamental distinction evident in the literature is between therapy‐oriented and non‐therapy‐oriented forms of dance. Therapy‐oriented forms, typically referred to as dance/movement therapy, or dance movement psychotherapy, are conceptualized as professional, relational practices delivered by registered therapists who use body movement and dance as therapeutic tools [46, 48, 49]. These forms emphasize the therapeutic relationship, the therapist's clinical training, and the intentional use of movement for psychological change. In contrast, non‐therapy‐oriented forms are generally led by dance or fitness instructors [46] or creative dance [44, 50] or may be self‐directed [51], with primary emphasis on physical activity, skill acquisition, and recreational engagement [46, 49, 52, 53, 54, 55].

Yet, these categories are neither mutually exclusive nor consistently defined [56]. For example, an exploratory dance session may be facilitated by either a therapist or a dance artist. This classificatory ambiguity has important implications. It limits comparability across studies, obscures which specific forms are most effective for which populations and outcomes, and hinders the development of practice guidelines. Synthesizing evidence across diverse dance forms, therefore, requires careful attention to how interventions are described and categorized in primary studies and reviews alike.

Amid this diversity and ambiguity, one structural commonality emerges across virtually all dance interventions: the session is typically organized into a phased, structured format. Regardless of whether the intervention is therapy‐oriented or recreational, most dance sessions follow a consistent temporal arc comprising warm‐up, main activity, and closure [57]. The warm‐up phase prepares participants physically and psychologically for engagement; the main activity constitutes the core dance practice, whether structured skill learning [58, 59], or improvisation [60]; and the closure phase provides an opportunity for cool‐down, reflection, or group sharing. Recognizing this structural consistency offers a useful counterpoint to the field's conceptual fragmentation. While the content and purpose of dance interventions vary widely, their shared temporal architecture provides a common ground for comparing interventions and identifying which components may be driving observed effects. Future research should, therefore, report not only the type of dance employed, but also how sessions are structured, enabling reviewers to move beyond crude categorizations toward more nuanced understandings of how specific intervention components relate to specific outcomes.

### 3.3 Conceptualization of Effectiveness and Healing

Across the included reviews, the terms “healing” or, more broadly, “effectiveness” are conceptualized in ways that reflect the underlying assumptions of different intervention traditions. A consistent distinction emerges between how healing is understood in therapy‐oriented versus non‐therapy‐oriented dance interventions.

In therapy‐oriented contexts, healing is predominantly framed as symptom reduction or clinical improvement. Reviews in this tradition assess outcomes such as decreased depression, anxiety, or psychological distress, using standardized clinical measures to quantify change [51, 54]. This conceptualization aligns with the medical and psychotherapeutic paradigms within which dance/movement therapy is often situated, positioning healing as movement from a pathological state toward normative functioning.

In contrast, non‐therapy‐oriented dance interventions conceptualize healing in broader, more experiential terms. Here, healing encompasses emotional restoration and enhancement [51], often operationalized as improvements in mood, affect, or subjective well‐being. A recurring construct in this literature is vitality, described as a state of renewed energy and capacity to engage fully in satisfying activities [61]. Related constructs include relaxation [62] and mind−body integration [43, 51], which capture a broader conception of healing that goes beyond symptom reduction to encompass embodied experience and quality of life. For example, Liao et al. [42] found that dance interventions significantly improved vitality among perimenopausal, menopausal, and postmenopausal women, with low heterogeneity across studies, suggesting that vitality may be a particularly robust and consistently defined construct within this domain.

However, most reviews treat statistically significant improvements as self‐evidently meaningful, without interrogating whether the observed changes translate into tangible differences in participants’ daily lives. As Ren and Xia [63] caution, more research is needed to determine whether the improvements documented in controlled studies are sufficiently noticeable to matter in real‐world contexts. For example, whether a reduction in depression scores enables a person to re‐engage with work, relationships, or activities they previously found meaningful. This gap between statistical and practical significance limits the translational value of the evidence base for practitioners, policymakers, and participants themselves.

### 3.4 Measurement Practices and the Invisibility of Dance‐Specific Processes

How mental health outcomes are measured and analyzed shapes what can be known about the effects of dance interventions. Across the included reviews, quantitative approaches constitute the predominant mode of inquiry in dance intervention research [56, 64, 65]. Most quantitative studies rely on standardized psychological scales to assess mental health outcomes. Commonly used instruments include the Physical Self‐Perception Profile (PSPP), General Well‐Being Schedule (GWB), Symptom Checklist‐90 (SCL‐90), Short Form Health Survey (SF‐36), and Self‐Rating Anxiety Scale (SAS) [43]. These scales offer the advantages of established psychometric properties, comparability across studies, and efficiency in administration. However, their widespread use also reflects and reinforces a particular conceptualization of mental well‐being as measurable psychological states, aligned with the medical and psychological paradigms within which much dance intervention research is situated.

Qualitative approaches, while less common, provide insights that quantitative methods cannot capture. These studies typically employ video observation [43, 66], as well as interviews and participant reflections, to explore the embodied, relational, and subjective dimensions of the dance experience. Qualitative methods are particularly valuable for understanding how participants experience healing, the processes, meanings, and contextual factors that quantitative scales necessarily aggregate and abstract. Across the qualitative components, findings generally converged in highlighting embodiment, emotional expression and regulation, social connection, self‐confidence, and meaning‐making as key mechanisms through which dance‐based interventions may influence well‐being. These findings complement quantitative evidence by helping to explain why improvements in psychological, social, and quality‐of‐life outcomes may occur. However, the qualitative evidence remains limited by small samples, heterogeneous intervention descriptions, inconsistent reporting of analytic procedures, and limited attention to negative or ambivalent participant experiences. Therefore, while qualitative findings enrich the interpretation of the umbrella review, they should be treated as explanatory and hypothesis‐generating rather than as definitive evidence of effectiveness. Future qualitative interview guides should more explicitly examine perceived bodily change, relational safety, cultural fit, barriers to participation, adverse experiences, and sustained impact. In addition, some primary studies incorporate both quantitative and qualitative evidence through mixed‐methods [43, 44, 56, 66, 67], offering a more comprehensive picture of intervention effects.

However, a notable omission in this measurement landscape is the limited use of movement‐sensitive and dance‐informed approaches to documenting how change occurs through movement. Despite dance being the intervention medium, most studies rely on psychological outcome scales rather than methods that can capture movement quality, bodily experience, expressive change, relational interaction, or participants’ own meanings of movement. This limitation suggests that dance is often treated primarily as a delivery vehicle for psychological change, rather than as an embodied, relational, and artistic practice that requires attention to its own processes. At the same time, addressing this gap does not mean simply adopting established movement analysis systems uncritically. Frameworks such as Laban Movement Analysis and the Kestenberg Movement Profile are historically and culturally situated, may privilege particular movement vocabularies, and may not be appropriate for all populations or practice contexts [68, 69, 70, 71, 72].

Paradoxically, despite the dominance of quantitative methods, the number of meta‐analyses in this field remains relatively small. Among the 90 reviews synthesized in this umbrella review, meta‐analyses accounted for only 10, while 24 were classified as mixed‐method reviews (Table 1). This apparent contradiction is explained by a more fundamental characteristic of the field. The literature is highly specialized; each review tends to focus on a specific population or a particular outcome, with limited overlap across studies. This specialization, while enabling deep dives into particular populations or interventions, also fragments the evidence base. Each review operates within its own narrowly defined domain, drawing from a limited pool of primary studies that meet its specific inclusion criteria. Consequently, even though quantitative studies abound, they are distributed across dozens of distinct research silos, and the number of studies available for meta‐analysis within any given silo remains small. The field thus finds itself in an ironic position. It has embraced the quantitative paradigm, yet its very success in generating specialized knowledge has, paradoxically, limited its capacity to produce the large‐scale meta‐analytic syntheses that the paradigm promises.

The underutilization of movement‐based methods compounds this problem. Even if meta‐analysis were more feasible within each silo, it would only tell part of the story. The field's limited capacity to answer questions about how dance interventions work, what they feel like to participants, and what aspects of the dance experience are most therapeutic leaves critical gaps in our understanding.

### 3.5 Evidence Patterns Across Populations

The populations targeted in dance intervention research reveal important patterns about the field's focus, biases, and gaps. Across the 90 included reviews, we examined the distribution of populations by clinical status, age, and gender, three dimensions that together illuminate whose mental health is being studied and whose remains marginalized. Across the review literature, the evidence base is socially and clinically uneven, with the strongest concentration in older adults, women, and clinical populations, especially cancer and depression.

By clinical status, clinical populations are studied more extensively than nonclinical populations. As shown in Table 2, most reviews focus on individuals with diagnosed conditions or specific clinical needs. This emphasis suggests that dance intervention research is, to a significant extent, oriented toward therapeutic intervention rather than general health promotion, a finding that aligns with the field's roots in dance/movement therapy and its application in healthcare settings. Among clinical populations, cancer and depression emerge as the two most extensively studied conditions.

**TABLE 2:** Summary of the population.

Author (year)	Specific population
Abu‐Odah et al. (2024) [46]	Cancer patients
Nelson et al. (2023) [76]	Cancer patients
Rudolph et al. (2018) [78]	Cancer patients
Li et al. (2025) [75]	Elderly cancer patients
Koch et al. (2019) [49]	Patients with schizophrenia
Chiang et al. (2019) [126]	Patients with schizophrenia
Ren and Xia (2013) [63]	Patients with schizophrenia
Aithal et al. (2021) [43]	Children with autism
Koch et al. (2019) [49]	Patients with cognitive impairment
Wang et al. (2021) [123]	Patients with cognitive impairment
Wu et al. (2021) [117]	Elderly with cognitive impairment
Sánchez‐Alcalá et al. (2023) [89]	Elderly with cognitive impairment
Koch et al. (2019) [49]	Patients with affective disorders
Allred (2023) [125]	Terminally ill patients
Amonkar et al. (2021) [44]	Patients on the autism spectrum
De Jesus et al. (2020) [54]	Patients on the autism spectrum
Barnish et al. (2023) [52]	Adults with primary anxiety disorder
Barnish et al. (2023) [52]	Adults with primary depressive disorder
Luo et al. (2024) [81]	Adolescents with depressive disorder
Kiepe et al. (2012) [79]	Patients with depressive disorder
Meekums et al. (2015) [80]	Patients with depressive disorder
Mo et al. (2025) [83]	Patients with depression in mainland China
Bell et al. (2025) [50]	Patients with dementia
Wang et al. (2021) [123]	Patients with dementia
Karkou et al. (2023) [48]	Patients with dementia
Dumitru (2025) [55]	Adults with intellectual disabilities
Takahashi and Kato (2023) [57]	Patients with intellectual disabilities
Fatkulina et al. (2021) [74]	Patients with breast cancer
Kiepe et al. (2012) [79]	Patients with breast cancer
Patel et al. (2025) [77]	Breast cancer survivors
Lee et al. (2025) [128]	Patients with neurological disorders
Jiménez et al. (2019) [129]	Patients with neurological disorders
Kiepe et al. (2012) [79]	Patients aged >14 with physical or mental illnesses
Niranjan et al. (2022) [138]	Patients with noninfectious lung diseases
Prudente et al. (2024) [82]	Elderly diagnosed with anxiety/depression
Yang et al. (2025) [139]	University students with depression
Tomaszewski et al. (2023) [141]	Adults with psychological trauma
Oepen (2025) [56]	Overweight/obese adults
Oepen (2025) [56]	Overweight/obese adolescents and children
Şanlı et al. (2025) [112]	Population with distorted body image
Búrány and Tarkó (2025) [95]	Adolescents (10–18)
Burkhardt and Brennan (2012) [96]	Adolescents (10–18)
Du et al. (2025) [65]	Adolescents (10–18)
Liu et al. (2022) [136]	Adolescents (10–18)
Rao et al. (2019) [84]	Adolescents (10–18)
Búrány and Tarkó (2025) [95]	Young adults (18–35)
Du et al. (2025) [65]	Young adults (18–35)
Fan et al. (2025) [92]	Menopausal women
Liao et al. (2025) [42]	Perimenopausal, menopausal, and postmenopausal women
Guo et al. (2020) [93]	University students
Olvera (2008) [94]	University students
Jaldin et al. (2025) [87]	Elderly
Noice et al. (2014) [98]	Elderly
Ou et al. (2022) [99]	Elderly
Ronzi et al. (2018) [100]	Elderly
Yoong et al. (2024) [101]	Elderly
Zhang et al. (2025) [102]	Elderly
Sánchez‐Alcalá et al. (2023) [89]	Elderly without cognitive impairment
Lavania and Ballal (2026) [132]	Survivors of childhood sexual abuse
Levine and Land (2016) [133]	Individuals with trauma
Liang and Bryant (2024) [104]	Women with interpersonal trauma history
Podolski et al. (2023) [88]	Elderly without dementia
Rao et al. (2019) [84]	Adults
Schwender et al. (2018) [85]	Adults
Son and Kwon (2024) [86]	Adults
Salihu et al. (2021) [121]	Adults without musculoskeletal disorders
Schwender et al. (2018) [85]	Children and adolescents
Tao et al. (2022) [140]	Children and adolescents
Sheppard and Broughton (2020) [90]	Individuals without prior health problems
Shim et al. (2021) [91]	Individuals without prior health problems
Allred (2023) [125]	Caregivers of terminally ill patients

Cancer patients constitute the single most frequently studied clinical group, with multiple reviews focusing on this population [46, 49, 73, 74, 75, 76, 77, 78]. The concentration of cancer research reflects the growing recognition of dance interventions as a supportive care modality for individuals navigating the physical and emotional challenges of oncology treatment and survivorship.

Depression is the second most studied clinical condition, appearing across multiple reviews that span different age groups and populations. These include reviews focused on adults with primary depressive disorder [52, 79, 80], adolescents with depressive disorder [81], older adults diagnosed with anxiety/depression [82], and patients with depression in specific geographic contexts [83]. The wide coverage of depression‐related research across age groups and population contexts reflects not only the global burden of depression but also growing recognition of dance as an accessible and potentially beneficial intervention for mood‐related difficulties.

Nonclinical populations, while less numerous than clinical populations, constitute a distinct and important strand of literature. These studies are oriented not toward symptom reduction or disease management, but toward health promotion, well‐being enhancement, and flourishing, a focus that reflects a different conceptualization of healing, one centered on positive functioning rather than the absence of pathology. Within this nonclinical literature, two broad categories of populations emerge. The first comprises generally healthy individuals across the lifespan, including healthy adults [84, 85, 86], older adults without cognitive impairment [87, 88, 89], and individuals without prior health problems [90, 91]. These studies position dance as a resource for maintaining or enhancing well‐being in populations not currently experiencing significant health challenges. The second category comprises life‐stage‐specific populations for whom dance may address transitional or developmental needs. These include perimenopausal, menopausal, and postmenopausal women navigating physical and emotional changes [42, 92]; university students facing academic and social pressures [93, 94]; and adolescents and young adults in formative developmental periods [65, 84, 95, 96, 97]. Notably, research on university students, especially postgraduate or doctoral students, remains sparse despite the potential of early intervention for establishing lifelong healthy movement and emotional regulation patterns.

By age, older adults constitute the most frequently studied population across both clinical and nonclinical categories. Reviews focusing exclusively or primarily on older adults appear most prominently (e.g., Jaldin et al. [87]; Noice et al. [98]; Ou et al. [99]; Podolski et al. [88]; Prudente et al. [82]; Ronzi et al. [100]; Sánchez‐Alcalá et al. [89]; Yoong et al. [101]; Zhang et al. [102]). This focus aligns with broader societal concerns about aging populations and the search for nonpharmacological interventions to support physical and cognitive health in later life.

By gender, a striking imbalance emerges; the overwhelming majority of participants across studies are female. Multiple reviews explicitly note that their included studies predominantly recruited women [45, 87, 91, 103], and several focus exclusively on female populations—breast cancer patients [74], women with interpersonal trauma histories [104], and perimenopausal, menopausal, or postmenopausal women [42, 92]. The only exception identified in this review is Amonkar et al. [44], who reported that approximately 74.8% of participants in dance interventions for autism spectrum disorder were male. This gender imbalance has significant implications. It means that the evidence base for dance interventions is, to a large extent, an evidence base about women's experiences, with limited understanding of how men experience and benefit from dance. As Moratelli et al. [105] observe, primary studies and reviews that explicitly examine gender differences are currently lacking.

### 3.6 Evidence Patterns Across Mental Health Outcomes

Dance‐based interventions have multidimensional effects on mental health, including both the enhancement of positive psychological functioning and the reduction of negative psychological symptoms. However, these effects are not consistently stable, and substantial differences can be observed across populations, intervention types, and outcome measures.

#### 3.6.1 Positive Dimensions of Mental Health

First, existing studies show that dance interventions have significant effects on various flourishing outcomes among general populations across cultures and age groups [45, 91, 106, 107], including well‐being [49, 50, 52, 55, 64, 90, 96, 108], life satisfaction [94], and psychological competence [84]. These effects are relatively stable in nonclinical populations, although high heterogeneity suggests that outcomes may depend on intervention type, implementation, and study design [45]. Furthermore, although a meta‐analysis by Li et al. [103] suggests that performative dance, such as square dance, may be more effective than other types of physical activity in improving well‐being, this conclusion is also associated with high heterogeneity. In clinical populations (e.g., individuals with Parkinson's disease), the evidence remains unclear [41].

Second, in terms of emotional regulation, dance interventions show broad positive effects across populations and age groups [109]. Both healthy individuals and clinical populations may experience improvements in emotional states [56, 66, 73]. However, findings remain inconsistent in certain groups. For example, results among cancer survivors are mixed [77], while evidence from adolescents and student populations generally supports positive effects despite some limitations in study quality [97, 110].

Furthermore, from a mechanistic perspective, dance interventions may promote mental health through embodied experience and social relational pathways. On the one hand, dance movement therapy can improve body image, self‐perception, and bodily awareness, with relatively consistent findings across studies [56, 111, 112]. On the other hand, dance activities can enhance social connection and interpersonal relationships and reduce loneliness, particularly among the elderly and women in menopausal stages [42, 75]. These psychosocial benefits may also occur regardless of the severity of depression [86]. However, for some outcomes, such as self‐esteem, although positive effects have been reported, the overall quality of evidence remains low, and conclusions should be interpreted with caution [99, 113].

#### 3.6.2 Negative Psychological Symptoms

Compared with positive outcomes, the effects of dance‐based interventions on negative psychological symptoms present a more complex and debated picture. First, depression is the most commonly studied outcome, but findings are highly inconsistent. Early quasi‐experimental studies suggested positive effects, but their reliability is limited due to methodological issues such as the lack of control groups [114]. Later randomized controlled trials improved methodological rigor, yet results remain mixed. Overall, meta‐analyses indicate that dance movement therapy has a moderate effect on depressive symptoms [49], and this effect has been observed across different age groups, cultural contexts, and both clinical and nonclinical populations [48, 76, 83]. However, these improvements are often limited to the intervention period and are difficult to maintain at follow‐up [52], and the optimal dosage of intervention remains unclear [47].

From the perspective of population differences, dance interventions appear to produce more stable improvements in depression among adolescents and adults, whereas findings in older adults are more controversial. Some studies report no significant effects [88, 115], while others suggest potential benefits in older adults without comorbidities or cognitive impairment [82, 89]. In clinical populations, results are also highly heterogeneous. For example, findings among cancer patients are inconsistent [73, 76], although positive effects have been observed in breast cancer patients and survivors [74, 77]. In contrast, evidence in Parkinson's disease is relatively consistent [53, 116], while effects in dementia or cognitively impaired populations appear limited [48, 117].

A more fundamental issue concerns whether dance interventions have effects that are specific to dance or primarily reflect the broader benefits of physical activity. Comparative evidence remains mixed. Some reviews suggest that dance does not consistently outperform other forms of physical activity in reducing depression [67, 118] and may rank below activities such as Tai Chi or yoga in certain populations [93]. However, a recent systematic review and network meta‐analysis by Noetel et al. [119] reported a large effect size for dance in reducing depressive symptoms compared with active controls, although the confidence in this estimate was limited because it was based on a small number of dance studies (*k* = 5). Therefore, the key issue is not whether dance is categorically superior or inferior to other forms of exercise, but under what conditions dance may produce distinctive benefits.

Second, dance interventions show potential benefits for anxiety across populations, but the certainty of evidence is limited. Overall, dance interventions demonstrate some positive effects in both clinical and nonclinical groups [111]. However, the strength of this effect varies across populations. In adolescents, the evidence is relatively consistent. Ritter and Low [120] and Burkhardt and Brennan [96] report that dance interventions are more effective for adolescents and adults than for children, and Du et al. [65] also found reductions in anxiety across educational levels. However, effects in primary school students are limited. In contrast, findings in older adults are inconsistent. Podolski et al. [88] report only small and nonsignificant improvements in anxiety among older adults without dementia, while Prudente et al. [82] report significant reductions but with high heterogeneity and very low certainty of evidence. Santos et al. [115] find that dance is effective for state anxiety but not for trait anxiety in older adults. This inconsistency suggests that anxiety outcomes may depend more on the match between the intervention and the population than on a universal effect. In clinical populations, findings are also mixed. Studies on cancer patients often report no significant effects [46, 73], while positive outcomes have been observed in breast cancer patients [74], with further inconsistency reported by Patel et al. Evidence in Parkinson's disease is more positive [116]. For specific life stage populations, dance interventions appear particularly beneficial for menopausal women. Fan et al. [92] report that dance is more effective than other forms of exercise, while Liao et al. [42] report significant effects with high heterogeneity. For emerging topics such as ecological anxiety, empirical evidence remains limited [62]. Overall, these findings suggest that anxiety reduction may rely more on the alignment between intervention and population than on general effectiveness.

Third, compared with depression and anxiety, the effects of dance interventions on stress are more consistent in general populations [94, 105, 121]. However, in clinical populations, findings remain inconsistent. Some meta‐analyses report no significant effects in cancer patients [73], while others find positive outcomes in breast cancer patients [74].

In addition, research on cognitive function mainly focuses on older adults and clinical populations, with mixed findings. Several reviews suggest that dance interventions may enhance cognitive functioning in older adults [87, 102, 110], and sustained engagement in dance has also been associated with cumulative cognitive gains over time [98]. However, in populations with dementia, dance movement therapy appears to have limited effects on cognitive outcomes [48].

Overall, dance‐based interventions show promising but uneven effects on mental health. Positive outcomes such as well‐being, emotional regulation, and social connection appear relatively stable, while effects on negative symptoms (e.g., depression and anxiety) are more heterogeneous and context‐dependent. These differences may be explained not only by population characteristics, intervention type, and methodological quality, but also by gender imbalance in existing studies and the lack of a detailed understanding of the internal mechanisms of dance interventions. In particular, the dominance of female samples may contribute to greater consistency in certain areas such as breast cancer research, while the relationship between specific movement features and psychological changes remains underexplored.

### 3.7 Moderators and Sources of Heterogeneity

First, intervention type is often considered the most direct moderator. However, existing evidence is limited in quantity and shows a confusing pattern. Cheng et al. [67] reported a counterintuitive finding that solo dance produced better outcomes than partnered dance. This contradicts the common assumption that partnered or group dance, with stronger social interaction, should be more beneficial. Cheng et al. [67] suggested that solo dance may allow greater freedom and help participants focus on bodily experience, whereas partnered dance may involve social and coordination pressure. Although this explanation is insightful, it has not been empirically tested, and the study itself did not further explore the underlying mechanisms. Huang et al. [122] found that aerobic dance had no significant effects on cognitive and mental health outcomes in older adults, which further suggests that the influence of intervention type may not only depend on the distinction between solo and partnered formats, but also on the specific form, intensity, and population fit of the dance. Apart from these studies, few reviews have systematically examined intervention type as a moderator. More importantly, most existing discussions remain at a superficial level of classification and do not identify the active components of different dance types. It remains unclear whether outcomes are driven by movement complexity, musical rhythm, social interaction, or other factors.

Second, intervention dosage is another moderator that has been discussed relatively widely, but the evidence is highly inconsistent. On the one hand, there is increasing evidence suggesting that more is not necessarily better. Cheng et al. [67] found that a larger total intervention dosage was associated with poorer outcomes. Liao et al. [42] reported that lower frequency, fewer than three sessions per week, was key for improving depression, while higher frequency reduced effectiveness. Li et al. [103] found that when the duration of dance intervention was less than 9 weeks, the positive effects on well‐being in middle‐aged and older adults showed lower heterogeneity, whereas interventions longer than 9 weeks showed greater variability. The meta‐analysis by Wang et al. [123] further supports this pattern. An 8‐month intervention showed smaller effects than a 3‐month intervention, and sessions conducted two to three times per week for 35 min were less effective than sessions conducted twice per week for 1 h.

On the other hand, evidence suggests that higher doses lead to better outcomes. Fan et al. [92] found that interventions shorter than 12 weeks produced moderate improvements in depression, while those longer than 12 weeks showed slightly stronger effects. Huang et al. [122] similarly reported that interventions longer than 12 weeks were more effective than shorter ones for improving cognitive and emotional outcomes in older adults. Salihu et al. [121] also found that at least 150 min of dance per week was associated with better outcomes. In addition, some studies report no relationship. Jaldin et al. [87] found that the positive effects of dance on cognitive function in older adults were not influenced by intervention dosage, although the authors noted that the small number of included studies requires cautious interpretation.

Faced with these three patterns, where higher dosage is better, higher dosage is worse, or dosage has no effect, an important question arises. Why do these inconsistencies occur? The answer may not lie in dosage alone, but in its interaction with other variables. Across studies, intervention type, population characteristics, outcome measures, and timing of assessment differ substantially. The effect of dosage may emerge or disappear depending on how it interacts with these factors. However, most reviews focus on single variables and lack the ability to examine such interactions. In addition, many dosage analyses are exploratory and conducted after the fact rather than based on predefined hypotheses, which limits their reproducibility.

Finally, the choice of measurement tools is another potential moderator, but it has received very little attention in existing reviews. Sánchez‐Alcalá et al. [89] provide a rare example. In their meta‐analysis, results were significant when depression in older adults was measured using the GDS‐15, but not significant when measured using the Hospital Anxiety and Depression Scale (HADS). They suggested that this difference may be due to the limited use of HADS in the included studies, which reduces statistical power. It remains unclear whether differences across measurement tools reflect true differences in intervention effects or systematic bias introduced by the instruments themselves. This issue is important because this field is dominated by psychological scales, and a wide range of instruments are used. If the choice of measurement tool itself is a source of heterogeneity and has been largely overlooked, then conclusions drawn from the existing evidence should be interpreted with greater caution.

## 4 Discussion

**FIGURE 2:**
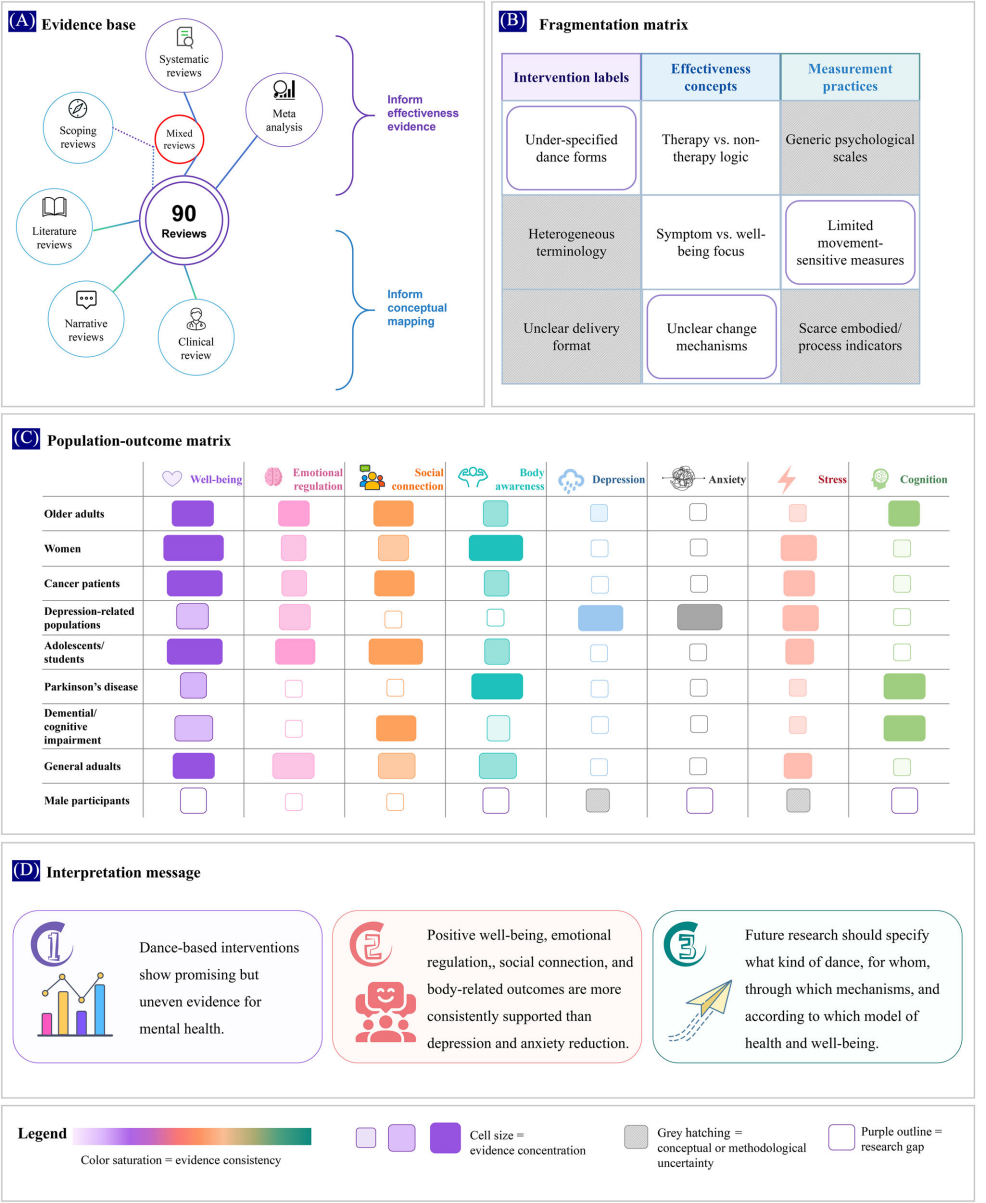
Interpretive framework of review‐level evidence on dance‐based interventions for mental health.

This quality‐sensitive umbrella review shows that the evidence on dance‐based interventions for mental health is fragmented not only because of methodological heterogeneity, but also because the field has not yet aligned three basic elements: what counts as dance, what counts as mental health change, and how such change should be measured. To synthesize these findings, Figure 2 presents an interpretive framework of the review‐level evidence. The framework integrates four layers: the composition of the evidence base, the fragmentation of intervention labels, effectiveness concepts, and measurement practices, the uneven distribution of population–outcome evidence, and the overall implications for future research. It should be read as a qualitative evidence map rather than a quantitative meta‐analytic summary.

Across the review literature, broad labels such as dance, dance therapy, dance/movement therapy, creative dance, social dance, and dance intervention are often used to describe practices that differ substantially in therapeutic intent, facilitator training, session structure, social format, and aesthetic orientation. Under these conditions, inconsistent findings should not be interpreted simply as evidence that dance‐based interventions are ineffective. Rather, they may reflect a field that has not yet developed sufficiently precise categories for asking what kind of dance is being used, for whom, through which processes, and according to which understanding of mental health.

### 4.1 From General Effectiveness to Specific Intervention Logic

A central implication of this review is that dance‐based interventions should not be treated as a single intervention category. Dance/movement therapy, square dance, ballroom dance, aerobic dance, creative dance, and community dance may all involve bodily movement, rhythm, and participation, but they are not theoretically or practically equivalent. Some are grounded in psychotherapy, some in exercise, some in social participation, and others in cultural or creative expression. Combining these forms under a general label of dance may increase the apparent breadth of the evidence base, but it also weakens interpretive precision.

The current evidence is, therefore, most meaningful when interpreted through specific population–intervention–outcome combinations. For example, therapist‐led dance/movement therapy for depressive symptoms, socially organized dance for loneliness among older adults, and creative dance for body awareness and emotional expression are more interpretable questions than whether dance works in general. Future studies and reviews should specify the form of dance, the delivery model, the facilitator's role, the intended process of change, and the outcome domain being targeted. Without this level of specification, both positive and null findings remain difficult to interpret.

### 4.2 Promising but Not Definitive Evidence

As summarized in Figure 2, the review‐level evidence suggests that dance‐based interventions show promise for selected mental health outcomes, especially positive well‐being, vitality, emotional expression, body awareness, social connection, and some short‐term symptom outcomes. However, this should not be taken as evidence for a stable general effect across all forms of dance and all populations. Many included systematic reviews and meta‐analyses were limited by weaknesses in protocol reporting, duplicate screening or extraction, risk‐of‐bias assessment, publication‐bias assessment, and the use of risk‐of‐bias information when interpreting results. These limitations do not invalidate the field, but they do mean that effectiveness claims should remain cautious. A further limitation concerns the review‐level nature of the present synthesis. Because this umbrella review synthesizes existing reviews rather than researching and reanalyzing all primary intervention studies, its findings necessarily reflect the evidence that has already been captured, classified, and emphasized in the review literature. In a fragmented field, some primary studies may be omitted from existing reviews, included under broad categories, or made difficult to trace because of inconsistent labels, populations, comparators, and outcome measures. As a result, some outcome patterns summarized here may be qualified, challenged, or contradicted by individual primary studies that have not yet been incorporated into review‐level syntheses. The findings should, therefore, be interpreted as a map of current review‐level evidence, rather than as a definitive adjudication of all primary evidence.

Positive functioning outcomes appear more consistently reported than reductions in depression and anxiety. This may be because outcomes such as social connection, vitality, emotional expression, and body awareness are more closely aligned with the experiential qualities of dance participation. By contrast, symptom outcomes may depend more strongly on baseline clinical severity, comparator type, intervention dosage, follow‐up timing, and population characteristics. The most defensible conclusion is, therefore, not that dance‐based interventions are universally effective, but that certain forms of dance may be beneficial for certain groups and outcomes under specific conditions.

### 4.3 Measurement Practices and Dance‐Specific Processes

A key finding of this review is that current measurement practices may obscure the distinctive value of dance‐based interventions. Most reviews rely heavily on generic psychological or clinical scales. These measures are useful for standardization and comparison, but they are less able to capture movement quality, bodily attunement, expressive transformation, aesthetic engagement, relational synchrony, or changes in embodied self‐experience. As a result, the field may be better equipped to detect broad exercise‐related effects than dance‐specific processes.

This helps explain why some reviews find that dance does not consistently outperform other forms of physical activity. Such findings may reflect true overlap between dance and exercise, but they may also reflect measurement limitations. If dance is expected to work through physical activation, generic mental health scales may be sufficient. If it is expected to work through emotional expression, body awareness, social synchrony, or aesthetic experience, then psychological scales alone are inadequate. Future studies should, therefore, combine standardized mental health measures with movement‐sensitive methods, qualitative accounts, process documentation, and culturally responsive approaches that attend to participants’ embodied experiences, expressive processes, and social contexts.

### 4.4 Future Directions

The next stage of research should focus less on accumulating broad evidence that dance works and more on aligning intervention form, proposed mechanism, outcome domain, and measurement strategy. Researchers should clearly describe the dance form, session structure, facilitator background, use of music, degree of improvisation, group format, participant choice, and reflection components. Reviews should also avoid treating all dance‐based practices as equivalent and should interpret findings according to review type, methodological quality, and likely primary‐study overlap.

This review also highlights the need for broader and more balanced population coverage. Current evidence is concentrated in older adults, women, cancer‐related populations, depression‐related populations, and selected neurological or cognitive conditions. These groups are important, but findings from them should not be generalized automatically to children, adolescents, men, gender‐diverse participants, nonclinical adults, students, or culturally diverse communities. Future research should examine how gender, age, culture, clinical status, and social context shape participation, acceptability, and outcomes.

Overall, the field of dance‐based interventions for mental health has moved beyond the simple question of whether dance is beneficial. The more important question is what form of dance is delivered, by whom, for which population, through which embodied, relational, aesthetic, or psychological processes, and measured according to which understanding of mental health. Answering this question requires not only more studies, but better specified, better measured, and more transparently synthesized evidence.

## 5 Conclusion

This quality‐sensitive umbrella review shows that the literature on dance‐based interventions for mental health has expanded substantially but remains fragmented across intervention definitions, outcome concepts, measurement practices, and review quality. The most credible evidence appears in specific population–intervention–outcome combinations rather than as a stable general effect across all forms of dance. Positive outcomes such as well‐being, emotional expression, social connection, and body‐related processes appear more consistently supported than reductions in depression or anxiety, but these findings remain constrained by inconsistent terminology, variable review quality, reliance on generic psychological measures, and limited attention to primary‐study overlap. Future research should move beyond asking whether dance works in general and instead align dance form, target population, proposed process of change, outcome domain, and measurement strategy. Strengthening the field will depend not only on producing more evidence, but on producing evidence that is conceptually clearer, methodologically more transparent, and better aligned with dance as an embodied, relational, and artistic practice.

## Author Contributions

X.D. contributed to the screening, coding, and writing of the manuscript. X.Z. assisted with screening and coding.

## Conflicts of Interest

The authors declare no conflicts of interest.

## Data Availability

No primary dataset was generated or analyzed in this study. The materials underlying this umbrella review, including the extracted summary information from the included studies, are publicly available in Figshare via the following https://doi.org/10.6084/m9.figshare.31856659.
